# Switching to Glycerol Phenylbutyrate in 48 Patients with Urea Cycle Disorders: Clinical Experience in Spain

**DOI:** 10.3390/jcm11175045

**Published:** 2022-08-28

**Authors:** Elena Martín-Hernández, Pilar Quijada-Fraile, Patricia Correcher, Silvia Meavilla, Paula Sánchez-Pintos, Javier de las Heras Montero, Javier Blasco-Alonso, Lucy Dougherty, Ana Marquez, Luis Peña-Quintana, Elvira Cañedo, María Concepción García-Jimenez, Pedro Juan Moreno Lozano, Mercedes Murray Hurtado, María Camprodon Gómez, Delia Barrio-Carreras, Mariela de los Santos, Mireia del Toro, María L. Couce, Isidro Vitoria Miñana, Montserrat Morales Conejo, Marcello Bellusci

**Affiliations:** 1 Centro de Referencia Nacional (CSUR) y Europeo (MetabERN) en Enfermedades Metabólicas, Hospital Universitario 12 de Octubre, Instituto de Investigación i+12, CIBERER, 28041 Madrid, Spain; 2 Centro de Referencia Nacional de Enfermedades Metabólicas (CSUR), Hospital La Fé de Valencia, 46026 Valencia, Spain; 3 Centro de Referencia Nacional (CSUR) y Europeo (MetabERN) de Enfermedades Metabólicas, Hospital San Joan de Deu Barcelona, 08950 Esplugues de Llobregat, Spain; 4 Centro de Referencia Nacional (CSUR) y Europeo (MetabERN) de Enfermedades Metabólicas, Hospital Clínico Universitario de Santiago de Compostela, IDIS, CIBERER, 15706 Santiago de Compostela, Spain; 5 Division of Pediatric Metabolism, CIBERER, MetabERN, Cruces University Hospital, University of the Basque Country (UPV/EHU) and Biocruces-Bizkaia Health Research Institute, 48903 Barakaldo, Spain; 6 Sección de Gastroenterología y Nutrición Infantil, Unidad de Enfermedades Metabólicas Hereditarias, Grupo IBIMA Multidisciplinar Pediátrico, Hospital Regional Universitario de Málaga, 29010 Málaga, Spain; 7 Centro de Referencia Nacional (CSUR) y Europeo (MetabERN) de Enfermedades Metabólicas, Hospital Vall D’Hebrón, 08035 Barcelona, Spain; 8 Unidad de Gastroenterología y Enfermedades Metabólicas, Hospital de Badajoz, 06002 Badajoz, Spain; 9 Unidad de Gastroenterología y Nutrición Pediátrica, Complejo Hospitalario Universitario Insular Materno-Infantil de Las Palmas, CIBEROBN, ISCIII, ACIP, Universidad de Las Palmas de Gran Canaria, 35016 Las Palmas de Gran Canaria, Spain; 10 Unidad de Gastroenterología y Nutrición, Hospital del Niño Jesús, 28009 Madrid, Spain; 11 Unidad de Metabolismo, Hospital Miguel Servet, 50009 Zaragoza, Spain; 12 Unidad de Enfermedades Musculares y Metabólicas Hereditarias, Departamento de Medicina Interna, Hospital Clinic, 08036 Barcelona, Spain; 13 Pediatría, Sección de Nutrición y Errores Innatos del Metabolismo, Complejo Hospitalario Universitario de Canarias, 38320 San Cristóbal de La Laguna, Spain

**Keywords:** clinical practice, glycerol phenylbutyrate (GPB), sodium benzoate (NaBZ), sodium phenylbutyrate (NaPB), urea cycle disorders (UCDs)

## Abstract

Background and objectives: Glycerol phenylbutyrate (GPB) has demonstrated safety and efficacy in patients with urea cycle disorders (UCDs) by means of its clinical trial program, but there are limited data in clinical practice. In order to analyze the efficacy and safety of GPB in clinical practice, here we present a national Spanish experience after direct switching from another nitrogen scavenger to GPB. Methods: This observational, retrospective, multicenter study was performed in 48 UCD patients (age 11.7 ± 8.2 years) switching to GPB in 13 centers from nine Spanish regions. Clinical, biochemical, and nutritional data were collected at three different times: prior to GPB introduction, at first follow-up assessment, and after one year of GPB treatment. Number of related adverse effects and hyperammonemic crisis 12 months before and after GPB introduction were recorded. Results: GPB was administered at a 247.8 ± 102.1 mg/kg/day dose, compared to 262.6 ± 126.1 mg/kg/day of previous scavenger (46/48 Na-phenylbutyrate). At first follow-up (79 ± 59 days), a statistically significant reduction in ammonia (from 40.2 ± 17.3 to 32.6 ± 13.9 μmol/L, *p* < 0.001) and glutamine levels (from 791.4 ± 289.8 to 648.6 ± 247.41 μmol/L, *p* < 0.001) was observed. After one year of GPB treatment (411 ± 92 days), we observed an improved metabolic control (maintenance of ammonia and glutamine reduction, with improved branched chain amino acids profile), and a reduction in hyperammonemic crisis rate (from 0.3 ± 0.7 to less than 0.1 ± 0.3 crisis/patients/year, *p* = 0.02) and related adverse effects (RAE, from 0.5 to less than 0.1 RAEs/patients/year *p* < 0.001). Conclusions: This study demonstrates the safety of direct switching from other nitrogen scavengers to GPB in clinical practice, which improves efficacy, metabolic control, and RAE compared to previous treatments.

## 1. Introduction

Urea cycle disorders (UCDs) are inborn errors of the metabolism caused by defects in any enzyme or transporter involved in the urea cycle, which converts ammonia to urea. This leads to ammonia and glutamine accumulation at toxic levels in the blood and brain of the affected patients [1]. The overall estimated incidence of UCDs in the United States is 1:35,000 births [2], and our previous study established a prevalence of 2.2 cases per one million inhabitants in Spain [3]. Clinical presentation depends on the defect type and deficiency severity; of particular clinical relevance are the severe UCDs present shortly after birth that involve hyperammonemic crisis (HAC), high mortality rates, cognitive impairment, and high risk of recurrence [4]. Treatments include a low protein diet (with arginine or citrulline supplementation) and nitrogen scavengers, while severe cases can benefit from liver transplantation [1,3,4,5].

Nitrogen scavengers routinely used include sodium benzoate (NaBZ), sodium phenylbutyrate (NaPB), glycerol phenylbutyrate (GPB), or their combination. They are salt or esters of benzoic acid (BA), phenylacetic acid (PAA), and phenylbutyric acid (PBA). NaBZ is conjugated with glycine to form hippurate that is excreted in the urine. NaPB and GPB are prodrugs of phenylbutyrate (PBA), which is metabolized in the liver to phenylacetyl-CoA, and then conjugated with glutamine to form phenylacetylglutamine (PAGln) and subsequently excreted in urine. [6]. NaPB is very effective in lowering ammonia, but with adherence problems due to taste, body odor, and dosing volume, in addition to high salt content [7].

GPB is the most recent molecule available for chronic UCDs management. It is a liquid triglyceride formulation for enteral use, which consists of three PBA molecules joined to a glycerol backbone. It is a virtually tasteless and odorless liquid, does not contain sugar or sodium, and is administered at low volume doses. Since GPB hydrolysis commences after it is exposed to pancreatic lipases in the small intestine, PBA orally delivered as GPB enters the circulation about 75% slower than NaPB. This slow PBA release might be associated with a clinically relevant benefit of GPB in comparison with NaPB, which results in more sustained ammonia control throughout the day [8,9].

GPB has demonstrated safety and efficacy in several patient cohorts by means of its clinical trial program [10,11,12,13,14,15,16,17]. Nowadays, GBP is approved for the chronic management of UCDs (those that cannot be managed by dietary protein restriction and/or amino acid supplementation alone) in newborns and older by the European Medicines Agency (EMA) and by the Food and Drug Administration (FDA) [18,19].

Although research with GPB has produced the largest amount of controlled study data in UCD patients to date, there are limited data on chronic GPB therapy in clinical practice [20,21]. The primary objective of this study was to analyze the efficacy and safety of GPB in clinical practice in Spanish UCD patients switching from another ammonia scavenger, during the first year of treatment.

## 2. Materials and Methods

### 2.1. Study Design

This was an observational, retrospective, multicenter study on Spanish UCD patients during their first year of treatment with GPB in standard clinical practice. The study was performed by the UCD study group of the Spanish Association of Inborn Errors of Metabolism (AECOM).

Inclusion criteria were patients with genetically defined UCD, switching from NaPB and/or NaBZ to GPB and administration of GPB treatment for at least one year. Patients who received any experimental treatment (gene therapy) or those who received liver transplantation were excluded.

Study data were collected and managed using REDCap electronic data capture tools hosted at Instituto de Investigación 12 de Octubre (imas12). The study was approved by both the Local Ethics Committee (protocol number 20/030, 9 June 2020) and by Spanish authority. The study protocol was approved by the local research Ethics Committee of each center involved and informed consent was obtained from every patient.

### 2.2. Endpoints and Variables

Data were collected from June 2019 to November 2021. Population characteristics included gender, diagnosis (carbamylphosphate synthetase 1, ornithine transcarbamylase, arginosuccinate synthetase 1, arginosuccinate lyase, or arginase 1 deficiencies), and presentation (neonatal, late onset, neonatal screening, familiar high-risk study). In all cases, switching was directly performed; GPB commenced and the previous scavenger was discontinued on the same day. Data related to drug switching were collected at three different times: the GBP switching day (T0); first evaluation after GPB introduction (T1); and one year after GBP treatment (T2).

At T0 and T2, the variables collected were age, anthropometric parameters (weight, height, cranial perimeter), dietary parameters (natural proteins, essential amino acids [EAA], total proteins), biochemical data (ammonia, glutamine, branched-chain amino acids [BCAA]), drug posology, number of hospital admissions because of hyperammonemic crisis (HAC), defined as the presence of clinical symptoms, such as nausea, vomiting, headache, and food intolerance, associated with a venous ammonia concentration ≥ 100 μmol/L) and number of visits to the emergency department in the last 12 months of treatment. To assess tolerance and security, related adverse effects (RAE) were recorded over both periods, including laboratory test to assess vital organ function. To assess adherence, urinary phenylacetylglutamine (U-PAG) levels (an excreted metabolite that correlates with nitrogen scavenger dose [22] were collected for both periods). Patient drug preference was also compiled (GPB or previous treatment). The data obtained at T1 only included both dietary and biochemical data and HAC.

Anthropometric parameters were collected by standard procedures in each center and expressed as z-scores, using published Spanish population values [23,24].

Laboratory assessments were performed at local certified laboratories. Samples for ammonia, glutamine and amino acids were collected after overnight fasting and prior to breakfast and first daily drug dose. To establish BCAA deficiency, the following normal range thresholds were used: leucine: <50 µmol/L, isoleucine < 30 µmol/L, and valine < 90 µmol/L in patients < 1 year or <130 µmol/L in children aged > 1 year and adults.

### 2.3. Statistical Analysis

All continuous and qualitative variables are shown as mean and SD and percentages, respectively. A paired *t*-test was used to analyze changes in biochemical or clinical continuous variables repeated across different time points (T0-T1-T2). To compare the mean of two samples, *t*-test or Mann–Whitney U-test were applied, depending on data statistical distribution. Linear regression test was performed to study the linear relationship between continuous variables. Statistical significance was established at *p* < 0.05. Analyses were performed using IBM SPSS v25.0 (SPSS Inc., Chicago, IL, USA).

## 3. Results

### 3.1. Demographics and Baseline Characteristics of the Series

Overall, 13 centers reported a total of 48 cases (29 females) from nine Spanish regions. The sample’s epidemiologic and clinical characteristics are shown in Table 1. Mean age at GPB treatment switching was 11.7 (SD: 8.2) years, divided into different age groups: 0–2 years (*n* = 3, 6.2%), 2–6 years (*n* = 10, 20.8%), 6–12 years (*n* = 15, 31.2%), 12–18 years (*n* = 15, 31.2%) and >18 years (*n* = 5, 10.4%). Only three patients were asymptomatic at diagnosis, two were detected by neonatal screening, and one by familiar study. The diagnosis was confirmed by molecular study, except in one case. Only two patients were previously treated with NaBZ, and the remaining patients (*n* = 46/48, 95.8%) were treated with NaPB.

### 3.2. Early Switching to GPB (T0 to T1)

In all cases, switching was performed directly, starting GPB and discontinuing the previous scavenger on the same day (T0). Clinical and biochemical monitoring was performed after a mean time of 79 ± 59 days (T1), while patients remained on the same diet and supplements. The starting dose of GPB at T0 was 247.8 ± 102.1 mg/kg/day versus 262.6 ± 126.1 mg/kg/day of the latest dose of the previous scavenger (*p* = 0.25). No patient presented clinical decompensation in the period between T0 and T1. We observed a statistically significant reduction of mean ammonia levels from 40.2 ± 17.3 to 32.5 ± 13.9 μmol/L (−18%, *p* < 0.001) and mean glutamine levels from 791.4 ± 289.8 to 648.6 ± 247.4 μmol/L (−19%, *p* < 0.001) (Table 2).

There were 12 patients with ammonia level above 50 μmol/L (65 ± 11 μmol/L) at T0, whereas only 4 at T1 (56 ± 7 μmol/L). In all of these patients but one ammonia decreased after the switch.

### 3.3. Comparative Study after One Year of GPB Treatment (T0 to T2)

#### 3.3.1. GPB Dosage

A significant reduction in GPB dose was observed with mean dose 247.8 ± 102.1 mg/kg/day at T0 and 228.6 ± 87.3 mg/kg/day at T2 (*p* = 0.013). Six patients reduced the number of doses from four to three a day.

#### 3.3.2. Anthropometric Values, Dietary Parameters and BCAA Levels

Mean weight, height, and cranial perimeter z-score did not change significantly during GPB treatment (Table 3). Prescribed protein did not change significantly in any age group and was higher in younger patients. In this regard, 62.5% of patients received 34% of the proteins as EAA (Table 3).

Regarding BCAA levels, mean leucine and valine levels were significantly higher at T2 (*p* = 0.038 and *p* = 0.002, respectively) and remain within normal limits in both the pediatric and adult population at T2 in all series. Isoleucine levels were not significantly different (*p* > 0.05), but within normal levels (Table 3).

Overall, 13/46 patients (28.3%) during the NaPB period and 5/48 patients during the GPB period (10.4%) had low levels of two or three BCAA. During the GPB period, patients with low BCAA levels had less proteins prescribed.

#### 3.3.3. Ammonia, Glutamine and HAC

A statistically significant reduction was observed for mean ammonia levels from T0 to T1 with a starting value of 40.2 μmol/L (SD: 17.3 μmol/L) and a decrease to 32.6 μmol/L (SD: 13.9 μmol/L) in T1 (*p* < 0.001). Values remained significantly lower at T2 (*p* < 0.001, Table 4). Regarding glutamine values, statistical significance was observed from T0 to T2 (*p* = 0.017, Table 4).

There were 10 patients (20.8%) who had 15 HAC during the last year on NaPB/NaBZ treatment, while only two patients (4.2%) had three HAC during the first year with GPB treatment, representing a 12-month HAC rate of 0.31 ± 0.68 and 0.062 ± 0.32, respectively (*p* 0.02). This reveals a statistical difference (Table 4).

**Figure 1:**
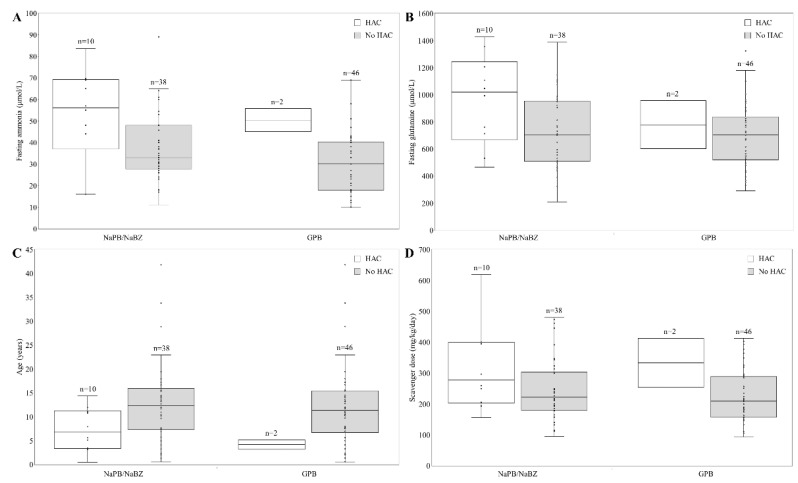
Characteristic of patient suffering hyperammonemic crisis (HAC). (**A**) Fasting ammonia levels (µmol/L) in patients with or without HAC treated with GPB or other nitrogen scavenger (NaPB/NaBZ). (**B**) Fasting glutamine levels (µmol/L) in those patients. (**C**) Age (years) of patients with or without HAC. (**D**) Scavenger dose received in patients with or without HAC. NaPB: sodium phenylbutyrate; NaBZ: sodium benzoate; GPB: glycerol phenylbutyrate.

Patients suffering HAC had significantly higher fasting ammonia and glutamine levels. Other parameters such as scavenger dose or age did not attain statistical significance (Figure 1).

There were 19 patients who visited the emergency room due to 43 episodes prior to switching (39.6%, rate 0.9, SD: 1.4) compared to 11 patients and 14 visits during GPB treatment (22.9%, rate 0.3, SD: 0.6, *p* = 0.004).

#### 3.3.4. RAE and Drug Preference

Only two RAE were reported with GPB treatment in two different patients: one with hair thinning and one with poor weight gain. No patient presented gastrointestinal problems, neurologic symptoms, or analytical abnormalities. Ten patients previously treated with NaPB presented 25 RAE, mostly gastrointestinal. Patients treated with NaBZ (*n* = 2/48) did not present RAE. Out of the total number of patients, the percentage suffering RAE treated with GPB was 4.1% (rate less than 0.1 RAE/patient/year) compared to 21.7% (rate 0.5 RAE/patient/year) during the period treated with other nitrogen scavengers (*p* = 0.04 for percentages and *p* < 0.001 for rates) (Table 5).

Regarding preference, 40 patients (83.3%) preferred GPB because of presentation form (*n* = 23, 57.5%), taste (*n* = 18, 45.0%), tolerance (*n* = 9, 22.5%), odor (*n* = 2, 5.0%), feeling better (*n* = 2, 5.0%), and/or effectiveness (*n* = 1, 2.5%). Only one adult patient preferred NaPB because of hair thinning with GPB. Patients previously treated with NaBZ preferred GPB.

#### 3.3.5. U-PAG

At T0, mean U-PAG values obtained in 15 patients were 14,803 µg/mL (SD: 9187), with three patients obtaining low values according to the thresholds proposed by Mokhtarani et al. [22] (<9000 μg/mL for <2 years old patients, <7000 μg/mL for >2 years with BSA ≤1.3 m^2^, and <5000 μg/mL for >2 years of age with BSA >1.3 m^2^). Mean U-PAG values in 12 patients at T2 were 16,099 µg/mL (SD: 8869), with only one patient with low values, who had an ammonia level of 18 µmol/L. In patients with both PAG values, consisting of a sample of 10 patients out of 48, mean U-PAG was 15,764 µg/mL (SD: 9.6) at T0 and 16.4 (SD: 9617) at T2. There were no statistically significant differences.

## 4. Discussion

Despite the high number of results obtained from GPB clinical development in UCD patients, the lack of clinical practice evidence in patients with chronic UCD highlights the need for clinical reports in this regard. In this case, we present the results obtained in 48 patients.

A direct switch from NaPB to a total daily dose of GPB mL equal to either sodium phenylbutyrate tablets (g) × 0.86 or sodium phenylbutyrate granules (g) × 0.81, is recommended in the summary of product characteristics [25]. In our study, following this process, no patient suffered an HAC during the early switch to GPB. Moreover, ammonia and glutamine values significantly decreased in the first follow-up visit; after one year of GPB treatment, when dose per kg further diminished because of patient weight gain, biochemical metabolic control continued to improve.

Patients switched from NaBZ should be considered as PBA naïve and, consequently, should be dosed accordingly, from 8.5 mL/m^2^/day to 7 mL/m^2^/day depending on body surface area [25]. Yeo et al. transitioned eight patients from NaBZ following these instructions and adjusting the dosage at follow-up visits according to the patient’s protein tolerance, required daily dietary protein intake, and their previous NaBZ dose. They detected that an appropriate GPB dose (closer to the patient’s needs) could be predicted based on the previous dose of NaBZ by using the formula GPB (mL/m^2^/day) = 0.0155 × NaBZ (mg/kg/day) + 3.7809 [21]. In our series, only two adult patients were transitioned from NaBZ with the same dose in mg/kg, which was lower than that recommended in naïve patients and similar to the dose adjusted according to Yeo’s formula. These patients did not experience any HAC and the ammonium values decreased after the change to GPB from 26 to 10 µmol/L in one patient and from 32 to 25 µmol/L in the other.

Dietary data in clinical trials were pooled from four studies in 45 adult and 49 pediatric UCD patients treated over 12 months with GPB. They were prescribed a low-protein diet, although with a greater intake than that established by the recommendations for UCD patients, without changes on protein tolerance [26]. The protein prescription in our patients was lower than in the trials and in the same way it did not change throughout the first year of GPB treatment. Despite this, mean BCAA levels remained within normal limits in both the pediatric and adult population and were higher than those obtained in clinical trials in children (2–17 years), despite the fact that their limits for considering BCAA deficiency were lower [14,15]. These results might be due to the fact that a high percentage of patients included in our study (60%) were receiving 30% of their proteins as EAA, which would decrease the ammonia load in the urea cycle since they contain less nitrogen than natural proteins. Moreover, the higher BCAA content would counteract the deficiency of these amino acids found in patients treated with PBA [27].

Regarding HAC, our results were consistent with clinical trial data, where the rate was 0.29 HAC patients/year, compared to 0.59 HAC patients/year during the pre-enrolment period with NaPB [28,29]. In our series, HAC was lower in both periods, especially during GPB treatment, with virtually no decompensation in patients. This might be accounted for the lower protein prescription and also because some data were collected during the COVID-19 pandemic, when other infectious diseases and the risk of decompensation were less common. Therefore, our experience suggests that GPB reduced HAC, which improves metabolic profile. This is consistent with the clinical experience of Leamle et al., with an adolescent suffering from ornithine transcarbamylase deficiency who underwent a significant reduction in ammonia and glutamine levels in plasma and an improved metabolic stability (from 8.7 hospitalizations per year to one hospitalization in seven months) after switching to GPB [20].

We also observed that patients with HAC had higher fasting ammonia levels (Figure 1), suggesting that UCD patients may benefit from tight ammonia control. Current UCD treatment guidelines indicate that ammonia should be kept within normal limits but providing limited guidance on the specific timing of blood draws or target levels. Lee et al., with more than 1000 samples from patients, reported that fasting ammonia correlated strongly with its daily exposure and with HAC rate. For patients with fasting ammonia levels < 0.5 ULN, 0.5 to <1.0 ULN, and ≥1.0 ULN (upper limit of normal), the probability of a normal daily level average value was 87%, 60%, and 39%, respectively; and 10.3%, 14.1%, and 37.0% of these patients experienced ≥1 HAC over 12 months [28]. Furthermore, Kent et al., analyzing data from 100 adult and pediatric UCD patients participating in four short-term switchover studies from NaPB to GPB and three 12-month safety extension studies with GPB, established that HAC risk was five to seven times higher in those reporting the highest fasting ammonia levels [29]. However, fasting glutamine correlated weakly with daily glutamine exposure and was not a significant HAC predictor [30]. In clinical practice, Yeo et al. established that glutamine levels were broadly unchanged after the switch [21]; however, plasma glutamine levels for the unstable patient reported by Laemle et al. decreased and were almost maintained within the reference range after starting GPB treatment [20]. In our experience, glutamine levels decreased with GPB treatment, and were higher in patients with HAC.

Adherence to treatment is key to attain metabolic stability, less HAC, better neurologic outcome, and better life quality. Lack of compliance and adherence with UCD medications or diet have been reported as a cause of acute HAC in 10% to 15% of patients [16]. Improved adherence has been reported in pediatric patients switched to GPB due to improved taste and dosage characteristics [31]. Since RAE presence also affects adherence, we observed a significant reduction in RAE after switching; and the high U-PAG levels during GPB treatment also suggest an excellent compliance [32,33]. Patients treated with previous scavengers have reported negative effects, such as poor tolerability, high burden of treatment, bad taste, and nausea with NaPB and gastritis with NaBZ [7,34]. The Canadian Organization for Rare Disorders (CORD) provided information from the feedback of 52 families (patients and parents) about GPB. GPB-experienced respondents reported more stable ammonia levels with GPB and no difficulties taking the drug. Most reported either no side effects or easily managed ones. CORD reported no discontinuations in patients taking GPB [35]. Our patients preferred GPB because of a liquid presentation, taste, smell, and gastrointestinal tolerance. Only an adult patient, who presented thinning of the hair, preferred NaPB, which is a new finding regarding GPB treatment.

Finally, it is important to point out the importance of individualizing the treatment in these patients, restricting proteins while maintaining sufficient intake for growth, adding citrulline and/or arginine supplements at enough doses to take advantage of the functioning part of the urea cycle, and adjusting the dose of GPB to that necessary to maintain metabolic stability and ammonium and glutamine in normal range. In this way, admissions for hyperammonaemic crises will decrease and the neurological prognosis will improve, all of which will improve the cost-effectiveness ratio.

The main limitation of this study is the short follow-up period and retrospective design. Longer-term studies will be necessary to confirm our results and to analyze cost effectiveness. It should also be considered that the study was performed during the COVID-19 pandemic.

## 5. Conclusions

This study demonstrates the safety of direct switching from other nitrogen scavengers to GPB in clinical practice, which improves efficacy, metabolic control and RAE when compared to previous treatments. This is crucial to achieve an improvement in neurologic outcomes and life quality.

## Figures and Tables

**Table 1:** Demographic and clinical data at baseline.

	Patients (*n* = 48)
**Gender, *n* (%)**	
Male	19 (39.6)
Female	29 (60.4)
**Age at treatment switching to GPB, years (SD)**	11.7 (8.2)
**Height, z-score (SD)**	−1.04 (1.5)
**Weight, z-score (SD)**	−0.34 (1.2)
**UCD diagnosis, *n* (%)**	
Ornithine transcarbamylase deficiency	27 (56.2)
Arginosuccinate lyase deficiency	9 (18.7)
Arginosuccinate synthetase1 deficiency	8 (16.6)
Carbamylphosphate synthetase1 deficiency	3 (6.2)
Arginase 1 deficiency	1 (2.1)
**UCD Onset, *n* (%)**	
Neonatal	19 (39.6)
Late	26 (54.2)
Asymptomatic (screening/high risk)	3 (6.2)
**Previous scavenger, *n* (%)**	
NaPB	46 (95.8)
NaBZ	2 (4.2)

GPB: glycerol phenylbutyrate; NaBZ: sodium benzoate; NaPB: sodium phenylbutyrate; UCDs: urea cycle disorders.

**Table 2:** Early switching: basal and first assessment after GPB introduction (T0-T1).

	NaPB/NaBZ	GPB	*p*
Dose, mg/kg/day (SD)	262.6 (126.1)	247.8 (102.1)	0.25
Ammonia, μmol/L (SD)	40.2 (17.3)	32.5 (13.9)	<0.001
Glutamine, μmol/L (SD)	791.4 (289.8)	648.6 (247.4)	<0.001

GPB: glycerol phenylbutyrate; NaBZ: sodium benzoate; NaPB: sodium phenylbutyrate.

**Table 3:** Anthropometric values, dietary parameters, and BCAA levels.

	*n*	T0	T2	*p*
**Weight, z-score (SD)**	48	−0.3 (1.2)	−0.4 (1.1)	NS
**Height, z-score (SD)**	47	−1.0 (1.4)	−1.0 (1.4)	NS
**Cranial perimeter, z-score (SD)**	13	−1.0 (0.9)	−0.7 (1.3)	NS
**Total proteins, g/kg/day (SD)**	48	0.8 (0.3)	0.8 (0.3)	NS
**Age group, years (SD)**				
<2	3	1.1 (0.3)	1.1 (0.1)	NS
2–5	10	0.9 (0.3)	1.0 (0.3)	NS
6–11	15	0.8 (0.3)	0.7 (0.2)	NS
12–17	15	0.7 (0.2)	0.8 (0.2)	NS
>18	5	0.7 (0.3)	0.7 (0.2)	NS
**EAA, g/kg/day (SD)**	30	0.3 (0.2)	0.3 (0.2)	NS
**BCAA levels,** **µmol/L (SD)**				
Leucine (50–150)		75.5 (25.3)	83.2 (22.6)	0.038
Isoleucine (30–85)		40.1 (15.8)	44.7 (15.3)	NS
Valine (130–300)		133.4 (36.0)	149.6 (36.1)	0.002

BCAA: branched chain amino acids; EAA: Essential amino acids; NS: no statistically significant differences.

**Table 4:** Ammonia, glutamine and hyperammonemic crisis.

	T0	T2	*p*
Ammonia, μmol/L (SD)	40.23 (17.29)	31.22 (14.83)	<0.001
Glutamine, μmol/L (SD)	791.42 (289.80)	700.35 (234.43)	0.017
HAC/year/patient (SD)	0.31 (0.68)	0.06 (0.32)	0.02

HAC: hyperammonemic crisis.

**Table 5:** Related adverse events.

	NaPB (*n* = 46)	GPB (*n* = 48)
**Gastrointestinal disorders**		
Abdominal pain	5	
Abdominal distension	4	
Vomiting	2	
Constipation	4	
Oral discomfort	2	
Dysgeusia	2	
Dyspepsia	1	
Decreased appetite	1	
**Other symptoms**		
Body odour	1	
Poor weight gain		1
Dizziness	1	
Hair thinning		1
**Laboratory abnormalities (including blood count and functions of vital organs)**		
Hypertransaminasemia	2	
**Total events**	25	2
**Total patients**	10 (21.7%)	2 (4.1%)

GPB: glycerol phenylbutyrate; NaPB: sodium phenylbutyrate.

## Data Availability

The data that support the findings of this study are available from the corresponding author upon reasonable request.
